# Effects of Gaseous Ozone on Microbiological Quality of Andean Blackberries (*Rubus glaucus* Benth)

**DOI:** 10.3390/foods10092039

**Published:** 2021-08-30

**Authors:** Sandra Horvitz, Mirari Arancibia, Cristina Arroqui, Erika Chonata, Paloma Vírseda

**Affiliations:** 1 Research Institute for Innovation & Sustainable Development in Food Chain, Campus Arrosadía, Public University of Navarre, 31006 Pamplona, Spain; cristina.arroqui@unavarra.es (C.A.); virseda@unavarra.es (P.V.); 2 Food Science and Engineering Faculty, Technical University of Ambato, Av. Los Chasquis y Rio Payamino, Ambato 180206, Ecuador; marancibias@uta.edu.ec (M.A.); erii_2404@hotmail.es (E.C.)

**Keywords:** blackberry, gray mold, pathogens, storage quality, ozone treatment

## Abstract

Andean blackberries are highly perishable due to their susceptibility to water loss, softening, mechanical injuries, and postharvest diseases. In this study, the antimicrobial efficacy of gaseous ozone against spoilage (mesophiles, psychrotrophs, and yeasts and molds) and pathogenic (*E. coli*, *S. enterica*, and *B. cinerea*) microorganisms was evaluated during 10 days of storage at 6 ± 1 °C. Respiration rate and mass loss were also determined. Ozone was applied prior to storage at 0.4, 0.5, 0.6, and 0.7 ppm, for 3 min. The best results were observed with the higher ozone dose, with initial maximum reductions of ~0.5, 1.09, and 0.46 log units for *E. coli*, *S. enterica*, and *B. cinerea*, respectively. For the native microflora, maximum reductions of 1.85, 1.89, and 2.24 log units were achieved on day 1 for the mesophiles, psychrotrophs, and yeasts and molds, respectively, and this effect was maintained throughout storage. In addition, the lower respiration rate and mass loss of the blackberries ozonated at 0.7 ppm indicate that this treatment did not induce physiological damage to the fruit. Gaseous O_3_ could be effective in maintaining the postharvest quality of blackberries throughout refrigerated storage but higher doses could be advisable to enhance its antimicrobial activity.

## 1. Introduction

Andean blackberries (*Rubus glaucus* Benth) are mostly cultivated in temperate and cold climates in South America and are usually consumed processed as pulp, jams, juices, and desserts [1]. Nonetheless, the interest in blackberries as fresh fruit has increased in the last years driven by the consumer’s interest in berries as sources of bioactive compounds and health benefits [2].

As non-climacteric fruit, blackberries must be harvested at full maturity when they have the best organoleptic and nutritional quality. At this stage, they are also more susceptible to mechanical injuries and microbial attacks, which impair their commercial quality and shorten the postharvest shelf-life [3,4]. One of the main postharvest diseases affecting blackberries is gray mold, caused by *Botrytis cinerea* Pers.: Fr. The contamination with this mold may occur in the field or during the harvest, postharvest, and storage period, even under refrigeration conditions, leading to important economic losses of this crop [5]. In addition, the contamination of berries with pathogens can result in foodborne illness outbreaks and thus, microorganisms such as *Escherichia coli* or *Salmonella* are of great concern for fruit growers and processors.

The most extended methods used to control postharvest decay and guarantee fresh produce safety are the application of synthetic fungicides, washing with chlorine-based sanitizers, and storage at low temperature. Yet, the risk of appearance of pesticide-resistant strains of pathogens and of environmental pollution caused by the residues, together with consumers’ demands to minimize chemical use, led to increased restrictions by marketing chains and regulatory agencies on agrochemical use, especially for postharvest applications [6,7].

Likewise, washing before retail is not recommended for delicate fruit such as berries, as their skin may be damaged easily [8]. Furthermore, it is now known that under commercial conditions, postharvest washing can present limited efficacy in fresh produce decontamination, or it can even lead to cross-contamination events between batches [9]. Finally, rapid cooling after harvest and constant cold storage are key factors in delaying microbial growth and extending the postharvest shelf-life of berries [10]. However, cold storage may be insufficient to prevent growth of mold, particularly on berries coming from fields with a high pathogen inoculum [3]. Therefore, the development of alternative sanitizing methods to prolong the storage life of blackberries after harvest is needed.

Conventional thermal treatments are effective in killing foodborne microorganisms, but they can negatively affect the quality of fresh produce. Thus, different individual or combined, physical (hot water, irradiation, UV-light, ozone, cold plasma), chemical (salts, organic acids, natural compounds), and biological treatments have been studied for decay control in fresh fruit and vegetables. 

One of these alternatives could be the use of gaseous O_3_, which has a high oxidant capacity and can be used for the inactivation of a wide range of microorganisms and for the degradation of chemical contaminants and off-odors in storage rooms [11]. There are several advantages related to the use of O_3_ as a sanitizer: it is produced on site and does not require storage, and its precursors are abundant and economically advantageous. In addition, it decomposes almost immediately to oxygen, presenting no safety concerns regarding the consumption of chemical residues [12]. In 2001, the US Food and Drug Administration (FDA) declared ozone to be a generally recognized as safe (GRAS) substance for the commercial use as a disinfectant and sanitizer in food handling [13]. Due to these circumstances, its application in food processing is considered an environmental-friendly technology and it is allowed by organic certification [14].

Due to the lack of a protective skin and berries’ surface roughness and sensitivity, gaseous treatments are preferred for these fruit [15]. Moreover, it is a common practice to pick berries straight into the punnets and prepare them in the field for retail. Thus, only gaseous ozone is applicable to them before the punnets are sealed and dispatched [16]. The antimicrobial activity of gaseous ozone against spoilage bacteria and fungi, and pathogens such as *E. coli* and *Salmonella* spp., has been studied in different berries including, among others, strawberries, table grapes, blueberries, blackberries, and raspberries.

Gaseous ozone contributed to reduce sour rot [17], the germination of *Botrytis cinerea* conidia, and the incidence and severity of gray mold during cold storage of table grapes [18]. Similarly, ozone proved efficacy in lowering decay in strawberries [19,20], raspberries [21,22], mulberries [23], blueberries [20,24], and blackberries [25]. On the contrary, after an initial microbial reduction, the exposure to gaseous ozone resulted ineffective in preventing decay after cold storage of strawberries [26], mulberries [27], and blueberries [28,29].

Even though the highest ozone doses often resulted in the highest antimicrobial activity, low concentrations of ozone are preferred in order to minimize exposure of workers to potentially hazardous concentrations of the gas and to reduce the risk of physiological damage to the treated produce [30]. In effect, as different fruit vary in their sensitivity to ozone, an optimal treatment should be established for each particular product. Previous research indicated that storage under continuous ozone prevented fungal decay and extended the shelf-life of blackberries [25]. However, to the best of our knowledge, to date, there are no previous reports dealing with the control of native microflora and inoculated pathogens on Andean blackberries by means of pre-storage treatments with gaseous ozone at low concentrations.

Therefore, the objective of this study was to investigate the effect of low gaseous ozone doses (0.4–0.7 ppm for 3 min) on the growth of native microflora (total aerobic mesophilic bacteria, psychrophiles, and yeast–mold) and inoculated pathogens (*E. coli*, *S. enterica*, and *B. cinerea*) on Andean blackberries during refrigerated storage. The respiration rate and mass loss of the fruit were also determined as indicators of possible physiological damage.

## 2. Materials and Methods

### 2.1. Plant Material

The plant material used for this study was Andean blackberries (*Rubus glaucus* Benth), hand-harvested in Tungurahua Province, Ecuador at maturity stage 4 (dark red), according to external color of the fruit and following the color chart of the Ecuadorian Quality Standard for fresh blackberries [31]. Immediately after harvest, 30 kg of fruit were transported to the Technical University of Ambato for analyses. Fruit that were uniform in size and color, sound, and free from blemishes and injuries were selected for the study.

### 2.2. Microbial Strains

The strains of *Escherichia coli* (ATCC 25922) and *Salmonella enterica* (ATCC 9842) were obtained from the American Type Culture Collection (Gaithersburg, MD, USA) and *Botrytis cinerea* was kindly provided by BioSeb Organics Ltd. (Ambato, Ecuador).

### 2.3. Preparation of Inoculum

A loopful of each stock bacterial culture was individually transferred into 30 mL of brain heart infusion (BHI) broth (Difco, Detroit, MI, USA) and incubated at 37 ± 2 °C for 24 h prior to experimentation. Cells were used when a concentration of 10^9^ CFU mL^−1^ was reached. Microbial concentration was determined according to the McFarland scale at 600 nm (OD600) [32]. An isolate of *B. cinerea* was cultured in a Petri dish on potato dextrose agar (PDA, Difco, Detroit, MI, USA). Streptomycin (1.0 mg mL^−1^) was added to the media to inhibit bacterial growth and the plate was incubated at 25 ± 1 °C for 7 days. The fungi spores were scraped with a sterile loop and diluted with sterile distilled water. Conidia were counted using a hemocytometer and the suspension was adjusted to 10^7^ CFU mL^−1^. 

### 2.4. Inoculation of Andean Blackberries

The blackberries were distributed in transparent polyethylene terephthalate (PET) plastic containers with perforated lids. The fruit was placed in a single layer, with each box containing 100 ± 5 g of fruit. The blackberries of each box were inoculated to reach 10^4^ conidia g^−1^ of *Botrytis cinerea* and 10^4^ CFU g^−1^ of *E. coli* and *S. enterica* by placing the inoculum on the surface of the fruit with a calibrated micropipette. Three containers/pathogen were prepared for each treatment and evaluation date.

In order to avoid the growth of native fungi and guarantee the growth only of the inoculated microorganism, before the inoculation with *B. cinerea*, the fruit were disinfected with an ethanol solution (70%, *v*/*v*) for 10 s, washed with sterile distilled water, and dried at room temperature. Inoculated blackberries were air dried for 1 h at room temperature (20 ± 2 °C) in a biosafety cabinet to allow the attachment of the microorganisms to the fruit surfaces. Thereafter, the fruit was stored under refrigeration (6 ± 1 °C) for 24 h until ozone treatment.

### 2.5. Ozone Treatment

**Figure 1:**
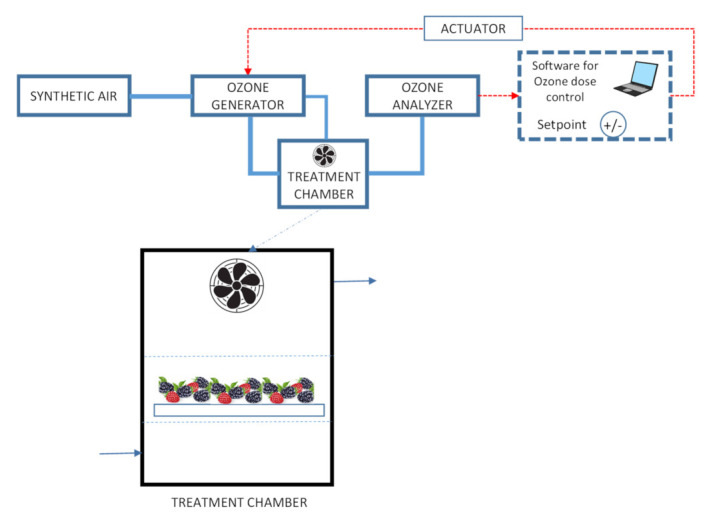
Diagram of gaseous ozone treatment system.

Ozone was generated in situ utilizing a surface discharge ozone generator (COM-SD-30, Anseros GmbH, Tübingen, Germany) and synthetic air as the feeding gas (Figure 1). A fan installed inside the treatment chamber (Precision, Pompano Beach, FL, USA) facilitated an even distribution of the gas. Ozone production was of 30 mg h^−1^ and ozone concentration was continuously monitored and controlled by circulating air from the chamber through an ultraviolet absorption ozone analyzer (Anseros MP; Anseros GmbH, Tübingen, Germany), calibrated in the range of 0–2000 ppm connected to a computer. The software integrated the concentration and time data and when the appropriate dose (concentration × time) was reached, the ozone generator was stopped.

Two independent ozonation processes were performed, one for the inoculated fruit and the second for the non-inoculated blackberries. In both cases, the fruit was divided in five groups: untreated berries represented the control sample, whereas the remaining four groups were subjected to gaseous ozone at 0.4, 0.5, 0.6, and 0.7 ppm, for 3 min.

#### 2.5.1. Inoculated Blackberries

The inoculated fruit was ozonized one day after the inoculation. For this purpose, the containers with the fruit were placed in the treatment chamber, with previous retirement of the lids. For each pathogen, three samples/evaluation date were treated with each of the ozone doses studied.

#### 2.5.2. Non-Inoculated Blackberries

The effect of ozonation on the native microflora of the blackberries was evaluated on non-inoculated samples. Around 2 kg of blackberries were placed on stainless steel mesh trays and taken inside the chamber for the treatment with each O_3_ dose.

### 2.6. Packing and Storage

After the O_3_ treatments, the fruit for the native microflora (aerobic mesophiles, psychrotrophs, and molds and yeasts) studies were packaged in the same transparent polyethylene terephthalate (PET) plastic containers (100 ± 5 g) as those used for the inoculated fruit. All the samples were stored at 6 ± 1 °C.

### 2.7. Respiration Rate

The respiration rate of the blackberries was measured using the closed system method [33]. Samples of 100 ± 10 g of blackberries were placed into glass jars with a hermetic closure and stored open at 6 ± 1 °C. On each evaluation date, the jars were closed and the internal O_2_ and CO_2_ were determined after 8 h with the use of an O_2_/CO_2_ gas analyzer (MAPY 4.0 LE SP, WITT-Gasetechnik, Germany). The results were expressed as mg of CO_2_ produced per kilogram per hour (mg kg^−1^ h^−1^).

### 2.8. Mass Loss

For mass loss, three trays were randomly selected and individually weighed at the beginning of the experiment, and every two days during the storage period. Results were expressed as percentage of mass loss relative to the initial mass.

### 2.9. Microbiological Analyses

Microbiological analyses were performed on days 1, 4, 7, and 10 of storage, considering day 1 as the day of ozonation. In addition, the containers were visually controlled daily in order to detect symptoms of microbial development. At each evaluation date, 3 samples/treatment were analyzed.

#### 2.9.1. Inoculated Microorganisms

For the analyses, 5 g of fruit inoculated with each of the pathogens under study was aseptically transferred to an individual filter stomacher bag and homogenized in 45 mL sterile buffered peptone water (Difco, USA) for 120 s at 200 rpm, using a Stomacher 400 circulator (Seward, AK, USA). Serial decimal dilutions of each homogenized sample were made in peptone water. From each dilution, 0.1 mL aliquots were aseptically surface-plated on the following media: Sabouraud dextrose agar plus chloramphenicol, Eosin Methylene Blue (EMB) Agar Levine, and Salmonella Shigella (SS) Agar, for *B. cinerea*, *E. coli*, and *S. enterica*, respectively. All the culture media were from Acumedia (Lansing, MI, USA). Culture conditions were as follows: 37 ± 2 °C for 48 h for *E. coli* and *S. enterica* and 25 ± 1 °C for 5 days in the case of *B. cinerea*.

#### 2.9.2. Native Microflora: Total Aerobic Mesophiles, Psychrotrophs, and Yeasts and Molds

Serial dilutions for these microbial groups were prepared as described above. From each dilution, 1 mL aliquots were aseptically pour-plated for mesophiles and psychrothrophs and 0.1 mL was surface-plated for molds and yeasts analyses. The following media and culture conditions were used: (1) plate count agar (PCA, Difco, Detroit, MI, USA) incubated at 35 ± 2 °C for 48 h and at 7 °C for 7 days, for total mesophilic and psychrotrophic microorganisms, respectively, and (2) Sabouraud dextrose agar plus chloramphenicol media (Acumedia, Lansing, MI, USA) incubated at 25 °C ± 2 for 5 days for yeasts and molds. All the samples were analyzed in duplicate, and microbial counts were expressed as log_10_ (cfu g^−1^) of fruit.

### 2.10. Statistical Analyses

The analyses were conducted in triplicate, considering each container as the experimental unit. Data were subjected to a one-way analysis of variance (α = 0.05) using the IBM SPSS Statistics Version 27 software (IBM Corporation, Armonk, NY, USA). When significant differences were observed, mean treatments were compared using Tukey’s test.

## 3. Results and Discussion

### 3.1. Respiration Rate

**Figure 2:**
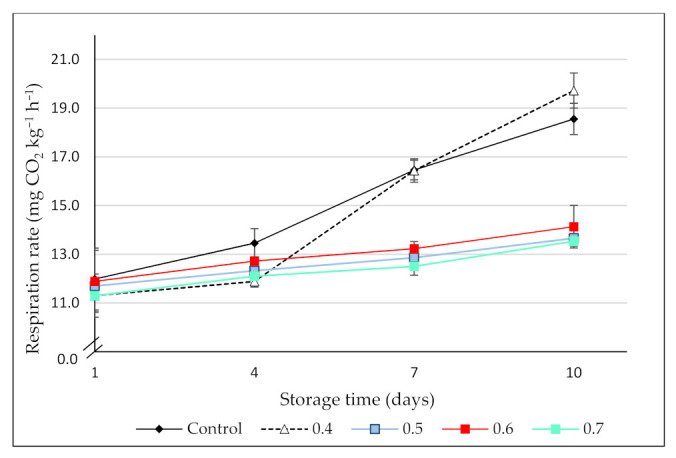
Respiration rate (mg CO_2_ kg^−1^ h^−1^) of control (no ozone treatment) and ozonated (0.4, 0.5, 0.6, and 0.7 ppm gaseous ozone/3 min) blackberries during 10 days of cold storage (6 ± 1 °C). Values are the mean of 3 independent samples and error bars represent the confidence interval (95%) for the mean.

Respiration rate (RR) is an indicator of the metabolic activity of fruit and vegetables and thus, an indicator of postharvest shelf-life. The RR of the control and the O_3_-treated blackberries is shown in Figure 2.

Initially, the respiration rate of the blackberries ranged between 11.30 ± 0.26 (0.4 ppm O_3_-treated blackberries) and 11.98 ± 0.51 (control) mg CO_2_ kg^−1^ h^−1^, with no significant differences among treatments (*p* > 0.05). Similar results were observed in cantaloupes [34], mulberries [23], and strawberries [35] treated with low doses of ozone. On the contrary, Forney et al. [36] reported an increase in the CO_2_ production of broccoli treated with high doses of this gas (7 ppm), which was attributed to physiological damage caused to the florets. During the storage period, the RR of all the fruit increased continuously. However, the highest CO_2_ production occurred in the control and those blackberries treated with 0.4 ppm O_3_. In effect, after 10 days, the RR of these fruit was significantly higher than in the blackberries ozonized with the highest doses. Chen et al. [34] and Han et al. [23] found similar inhibitory effects of ozone on respiration rate during storage of melons and mulberries, respectively. The elevated CO_2_ production observed in the present study, which represented an increase of around 55% and 75%, in the control and the 0.4 ppm O_3_-treated fruit, respectively, could be related to the highest microbial growth observed in these treatments by the end of the storage period.

### 3.2. Mass Loss

**Figure 3:**
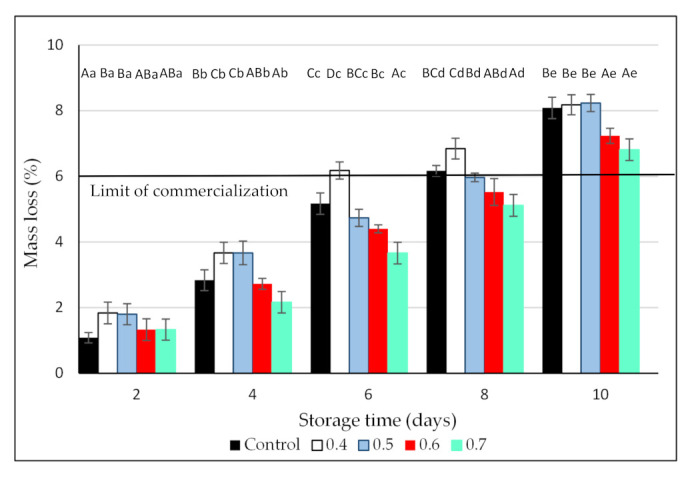
Effect of 3 min exposure to ozone (0, 0.4, 0.5, 0.6, and 0.7 ppm) on mass loss of Andean blackberries during 10 days of cold storage. Values are the mean of 3 independent samples and error bars represent the confidence interval (95%) for the mean. For each evaluation date, different capital letters indicate significant differences among O_3_ doses (*p* < 0.05). For each O_3_ dose, different lower-case letters indicate significant differences among evaluation dates (*p* < 0.05).

During the postharvest period, mass loss is caused mainly by the respiration and transpiration of the fruit [37]. In the case of blackberries, their high respiration rates together with the lack of a protective peel make these fruit very susceptible to moisture loss. In effect, regardless of the treatment, mass loss increased constantly during storage, with maximum values of around 8% after 10 days of refrigerated storage (Figure 3). This value is above the maximum mass loss acceptable for commercialization of blackberries, reported as 6% [38]. Nevertheless, in all the evaluation dates, the exposure of blackberries to the highest O_3_ dose resulted in lower mass loss, indicating that no physiological damage occurred due to ozonation. The lower mass loss in the fruit treated with the highest O_3_ concentrations could be associated with the lower respiration rates and microbial counts observed in these blackberries. Similar results were reported for strawberries [39] and winter jujubes [40] washed with aqueous ozone and in table grapes [41], red peppers [42], and blueberries [28] exposed to gaseous ozone. According to Contigiani et al. [6], a thicker and reinforced cuticle in the O_3_-treated fruit, which contributes to keeping cell integrity and offers a protective effect against moisture loss, can explain the positive effect of ozone in hindering mass loss.

### 3.3. Microbiological Analyses

#### 3.3.1. *E. coli* and *S. enterica*

The counts for *E. coli* and *S. enterica* in the blackberries as affected by the exposure to gaseous O_3_ and storage time at 6 °C are presented in Table 1.

Immediately after exposure to ozone, the counts of *E. coli* and *S. enterica* were significantly reduced in all the ozone-treated blackberries, with the greatest reductions observed with the highest O_3_ dose: 0.48 and 1.09 log units for *E. coli* and *Salmonella*, respectively. Ozone’s antimicrobial activity is based on its oxidant potential, which provokes injuries to the cell walls and a progressive oxidation of the microorganisms’ cellular components [43]. However, it has limited penetration and thus, can be ineffective against latent infections, microbial growth occurring in wounds, and bacteria attached to uneven surfaces of fresh produce, all of which restrict the contact of ozone with the target microorganisms [44]. In this sense, the low microbial reductions observed in this study could be explained by the roughness and irregularities of the blackberries’ surface where the inoculated bacteria can remain protected from ozone action and the relatively low O_3_ doses used. Similar findings were reported for ground pepper [45], raspberries and strawberries [46], and mushrooms [47] treated with gaseous ozone and demonstrate that surface area is critical regarding the efficiency of O_3_ treatments. Under these circumstances, higher O_3_ concentrations and/or longer exposures to the gas were necessary to achieve the pathogens’ inactivation.

During the storage period, the counts for both pathogens progressively decreased in all the treatments. The final counts of *E. coli* ranged from 2.96 ± 0.11 (0.7 ppm O_3_) to 3.49 ± 0.06 log units in the control samples, whilst *Salmonella* was not detected on day 10 in the blackberries exposed to 0.6 and 0.7 ppm O_3_. Daş et al. [48] reported similar findings in O_3_-treated cherry tomatoes inoculated with *Salmonella* after 6 days of cold storage. Both blackberries and tomatoes are acidic fruit and *Salmonella* is very susceptible to acid environments. On the contrary, some strains of *E. coli* remained viable even at pH 2.5 [49]. In this study, and in addition to ozone activity, the low pH (3–3.9) of the blackberries could contribute to limit the growth of this pathogen [50].

#### 3.3.2. *Botrytis cinerea*

**Figure 4:**
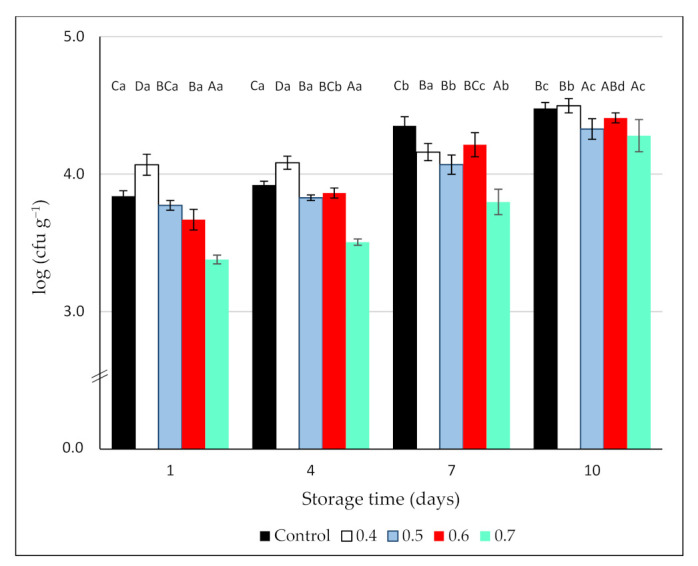
Effect of 3 min exposure to ozone (0, 0.4, 0.5, 0.6, and 0.7 ppm) on *Botrytis cinerea* counts (log (cfu g^−1^)) on inoculated blackberries during 10 days of cold storage. Values are the mean of 3 independent samples and error bars represent the confidence interval (95%) for the mean. For each evaluation date, different capital letters indicate significant differences among O_3_ doses (*p* < 0.05). For each O_3_ dose, different lower-case letters indicate significant differences among evaluation dates (*p* < 0.05).

The counts of *Botrytis* on non-ozonated blackberry samples or O_3_-treated fruit (0.4, 0.5, 0.6, and 0.7 ppm gaseous O_3_/3 min) are shown in Figure 4. On day 1, *Botrytis* counts for the control reached 3.84 ± 0.05 log units and the maximum reduction (0.46 log units) was observed in the blackberries treated with the highest O_3_ dose. In effect, only this treatment significantly reduced *Botrytis* counts initially and following the storage period (Figure 4). On the contrary, the treatment with 0.4 ppm O_3_ was not effective against this pathogen, with higher counts than the control. During the cold storage period, the growth of *Botrytis* progressively increased, and mycelium could be visually detected in the samples from all the treatments.

The results found in the literature regarding O_3_ efficacy to control *B. cinerea* are somehow contradictory. Vlassi et al. [51] found that, regardless of inoculation technique (injection or immersion), treating table grapes with gaseous ozone (15 ppm/60 min) on a daily basis was an effective means of controlling *Botrytis cinerea* during 40 days of cold storage. On the contrary, Sharpe et al. [29] reported that low doses of ozone (450 ppb applied for 48 h before storage) reduced decay incidence in apples and grapes but were ineffective in blueberries, probably due to the inability of ozone to penetrate the fruit tissue to kill latent infections occurring early in the growing season. In the same way, gray mold was controlled by ozone in table grapes but not in citrus or stone fruit [52]. These authors attributed the differences to the fact that in the former, the inoculum was on the surface of the fruit, whilst in the latter, the pathogen was inoculated into wounds, hindering the access of the ozone to the inoculum. Finally, in naturally infected fruit, the treatment with 0.15 (grapes) and 0.7 (strawberries) ppm gaseous ozone, applied continuously in the storage room completely inhibited *Botrytis* development [30]. These apparent discrepancies among studies can be explained by differences in the type of commodity, cultivars, the inoculum level, and the presence of wounds, as well as experimental conditions, such as gas concentration and time of exposure; factors that can influence the antimicrobial efficacy of ozone treatments [15].

#### 3.3.3. Native Microflora: Total Aerobic Mesophiles, Psychrotrophs, and Molds and Yeasts

The microbial counts for the native microflora of the control and the O_3_-treated blackberries are shown in Table 2. On the initial day, the control samples presented 2.93 ± 0.03 (aerobic mesophiles), 4.44 ± 0.15 (psychrotrophs), and 5.25 ± 0.09 (molds and yeasts) log unit counts. 

In all the O_3_-treated fruit, the counts for the three microbial groups studied were significantly lower (*p* < 0.05) when compared with the control. Furthermore, the extent of the reductions increased with increasing O_3_ dose, ranging from 0.27 to 1.85, 0.49 to 1.89, and 0.37 to 2.24 log units for the mesophiles, psychrotrophs, and molds and yeasts, respectively. During the cold storage period, the microbial populations gradually increased regardless of the treatment. Yet, in all the ozone-treated blackberries, the reductions achieved were maintained throughout the storage period. Among the O_3_ treatments, the best results were observed when the blackberries were exposed to 0.7 ppm O_3_ with counts on day 10 for total aerobic mesophiles and psychrotrophs even lower than the initial counts of the control samples.

Gaseous ozone can be applied either as a pre-storage treatment or continuously or intermittently during the storage period. Similar to our results, the application of gaseous ozone at doses of 130 g m^−3^/30 min and 0.5 to 2 mg L^−1^/60 min reduced the total mesophile counts in juniper berries [53] and greenhouse tomatoes [54], with the inhibitory effects being maintained during storage. In the same way, Alves et al. [55] found that 18 and 14 mg L^−1^ gaseous ozone applied to strawberries for 30 min were effective in controlling aerobic mesophiles and molds and yeasts, respectively, during 4 days of storage. In studies involving the application of ozone during storage, the exposure of sweet cherries to 2 ppm gaseous ozone for 30 min every 6 days delayed decay development and lowered decay incidence on the fruit for up to 18 days [56]. As well, treating raspberries with 8–10 ppm O_3_ in cycles of 30 min once every 12 h reduced the aerobic mesophilic bacteria and fungi counts on this fruit during 3 days of storage at room temperature [22]. On the contrary, both intermittent (0.1 ppm applied every 30 min) and continuous (0.35 ppm, 3 days) O_3_ applications were only partially effective in preventing fungal growth on table grapes and strawberries, respectively [26,57].

These variable and somehow contradictory results could be explained by differences in the type of product, differences in the ozone application methods and in the doses used (time and concentration), the microbial type and microbial load, as well as variations in environmental conditions (temperature, relative humidity), all of which can affect the efficacy of ozone as a sanitizer [58].

## 4. Conclusions

The application of low doses of gaseous ozone prior to storage was studied as an environmental-friendly alternative to guarantee Andean blackberries’ quality and safety. The best results were obtained after the exposure of the fruit to 0.7 ppm gaseous O_3_ for 3 min. This treatment slowed down the respiration and mass loss rates, indicating that no physiological damage occurred in the treated fruit. Moreover, it was proven effective in reducing both the native microflora and the inoculated pathogens on the fruit throughout the storage period. Therefore, gaseous ozone could be considered a promising processing technology for prolonging the postharvest life of fresh Andean blackberries during refrigerated storage. However, as the log reduction in microbial populations observed in this study may not be enough to ensure the safety of the product, further studies would be necessary to determine the optimal treatment conditions for this fruit. As well, the effects of the treatment on the bioactive compounds and the physicochemical and sensory quality of the blackberries would be assessed.

## Figures and Tables

**Table 1:** *E. coli* and *S. enterica* counts (log cfu g^−1^) of control (no O_3_ treatment) and ozonated (0.4, 0.5, 0.6, and 0.7 ppm gaseous ozone/3 min) blackberries during 10 days of cold storage (6 ± 1 °C).

Microorganism	Day	O_3_ Concentration (ppm)				
		0.0	0.4	0.5	0.6	0.7
*E. coli*	1	4.71 ± 0.02 Dd	4.45 ± 0.02 Cd	4.36 ± 0.05 BCd	4.27 ± 0.06 ABc	4.23 ± 0.13 Ac
	4	4.39 ± 0.05 Cc	4.30 ± 0.02 Bc	4.20 ± 0.08 BAc	4.19 ± 0.04 BAc	4.29 ± 0.04 Bc
	7	4.00 ± 0.02 Bb	3.99 ± 0.01 Bb	3.95 ± 0.03 BBb	3.92 ± 0.06 BBb	3.65 ± 0.09 Ab
	10	3.49 ± 0.06 Ba	3.39 ± 0.08 Ba	3.51 ± 0.08 BBa	3.05 ± 0.09 BAa	2.96 ± 0.11 Aa
*S. enterica*	1	4.47 ± 0.03 Ed	4.29 ± 0.09 Dd	3.96 ± 0.08 BCd	3.75 ± 0.10 BBd	3.38 ± 0.02 Ad
	4	4.00 ± 0.08 Cc	3.90 ± 0.03 Bc	3.87 ± 0.03 BBc	2.97 ± 0.10 BAc	2.98 ± 0.01 Ac
	7	3.25 ± 0.05 Eb	2.99 ± 0.19 Db	2.15 ± 0.05 BCb	1.04 ± 0.07 BBb	0.80 ± 0.06 Ab
	10	1.58 ± 0.14 Da	0.95 ± 0.16 Ca	0.50 ± 0.00 BBa	0.00 ± 0.00 BAa	0.00 ± 0.00 Aa

Values are the mean ± standard deviation (*n* = 3). For each microorganism and evaluation date, different capital letters indicate significant differences among O_3_ doses (*p* < 0.05). For each microorganism and O_3_ dose, different lower-case letters indicate significant differences among evaluation dates (*p* < 0.05).

**Table 2:** Aerobic mesophiles, psychrotrophs, and molds and yeasts counts (log cfu g^−1^) of control (no O_3_ treatment) and ozonated (0.4, 0.5, 0.6, and 0.7 ppm gaseous ozone/3 min) blackberries during 10 days of cold storage (6 ± 1 °C).

Microbial Group	Day	O_3_ Concentration (ppm)				
		0.0	0.4	0.5	0.6	0.7
Aerobic mesophiles	1	2.93 ± 0.03 Ea	2.66 ± 0.10 Da	2.25 ± 0.06 Ca	1.30 ± 0.19 Ba	1.08 ± 0.12 Aa
	4	3.23 ± 0.10 Cb	2.75 ± 0.16 Ba	2.54 ± 0.21 Bb	2.18 ± 0.03 Ab	2.08 ± 0.08 Ab
	7	4.00 ± 0.06 Dc	3.17 ± 0.02 Cb	2.58 ± 0.11 Bb	2.26 ± 0.12 Ab	2.29 ± 0.06 Ac
	10	4.00 ± 0.10 Ec	3.88 ± 0.02 Dc	3.66 ± 0.04 Cc	3.15 ± 0.07 Bc	2.73 ± 0.02 Ad
Psychrotrophs	1	4.44 ± 0.15 Ca	3.95 ± 0.12 Ba	2.70 ± 0.01 Aa	2.58 ± 0.02 Aa	2.55 ± 0.04 Aa
	4	4.54 ± 0.06 Da	4.07 ± 0.04 Cb	3.72 ± 0.02 Bb	4.15 ± 0.12 Cb	3.46 ± 0.25 Ab
	7	5.10 ± 0.03 Db	4.84 ± 0.00 Cc	4.29 ± 0.01 Bc	4.32 ± 0.23 Bb	4.01 ± 0.01 Ac
	10	6.18 ± 0.05 Ec	5.32 ± 0.07 Dd	4.89 ± 0.05 Cd	4.78 ± 0.03 Bc	4.11 ± 0.03 Ac
Molds and yeasts	1	5.25 ± 0.09 Ea	4.88 ± 0.05 Da	4.21 ± 0.03 Ca	3.22 ± 0.08 Ba	3.01 ± 0.17 Aa
	4	6.18 ± 0.12 Eb	5.30 ± 0.02 Db	5.04 ± 0.02 Cb	4.61 ± 0.03 Bb	4.36 ± 0.03 Ab
	7	6.81 ± 0.10 Ec	6.16 ± 0.01 Dc	5.60 ± 0.04 Cc	5.43 ± 0.02 Bc	5.31 ± 0.01 Ac
	10	7.07 ± 0.04 Dd	6.22 ± 0.00 Cd	6.20 ± 0.03 Cd	6.13 ± 0.02 Bd	5.79 ± 0.02 Ad

Values are the mean ± standard deviation (*n* = 3). For each microorganism and evaluation date, different capital letters indicate significant differences among O_3_ doses (*p* < 0.05). For each microorganism and O_3_ dose, different lower-case letters indicate significant differences among evaluation dates (*p* < 0.05).

## Data Availability

Not applicable.

## References

[1] Martínez A., Beltrán O., Velastegui G., Ayala G., Jácome R., Yánez M., Luciano E. (2007). Manual del Cultivo de la Mora de Castilla.

[2] Clark J.R., Finn C.E. (2014). Blackberry cultivation in the world. Rev. Bras. Frutic..

[3] Gabioud Rebeaud S., Varone V., Vuong L., Cotter P.Y., Ançay A., Christen D. (2020). Postharvest ozone treatment on raspberries. Acta Hortic..

[4] Horvitz S., Chanaguano D. (2020). Microbial and sensory quality of an Andean blackberry (*Rubus glaucus* Benth) cultivar. Acta Hortic..

[5] Junqueira-Gonçalves M.P., Alarcón E., Niranjan K. (2016). The efficacy of potassium sorbate-coated packaging to control postharvest gray mold in raspberries, blackberries and blueberries. Postharvest Biol. Technol..

[6] Contigiani E.V., Jaramillo-Sánchez G., Castro M.A., Gómez P.L., Alzamora S.M. (2018). Postharvest Quality of Strawberry Fruit (*Fragaria × Ananassa* Duch cv. Albion) as Affected by Ozone Washing: Fungal Spoilage, Mechanical Properties, and Structure. Food Bioproc. Tech..

[7] Romanazzi G., Smilanick J.L., Feliziani E., Droby S. (2016). Integrated management of postharvest gray mold on fruit crops. Postharvest Biol. Technol..

[8] Vardar C., Ilhan K., Karabulut O.A. (2012). The application of various disinfectants by fogging for decreasing postharvest diseases of strawberry. Postharvest Biol. Technol..

[9] Murray K., Wu F., Shi J., Jun Xue S., Warriner K. (2017). Challenges in the microbiological food safety of fresh produce: Limitations of post-harvest washing and the need for alternative interventions. Food Qual. Saf..

[10] Horvitz S., Chanaguano D., Arozarena I. (2017). Andean blackberries (*Rubus glaucus* Benth) quality as affected by harvest maturity and storage conditions. Sci. Hortic..

[11] Torlak E. (2019). Use of gaseous ozone for reduction of ochratoxin A and fungal populations on sultanas. Aust. J. Grape Wine Res..

[12] Pandiselvam R., Sunoj S., Manikantan M.R., Kothakota A., Hebbar K.B. (2017). Application and Kinetics of Ozone in Food Preservation. Ozone Sci Eng..

[13] United States Food and Drug Administration (2001). Rules and Regulations. Part 173-Secondary Direct Food Additives Permitted in Food for Human Consumption (21 CFR Part 173 Authority: 21 USC. 321, 342, 348).

[14] Zainuri J., Sauqi A., Sjah T., Desiana R.Y. (2018). Combination of ozone and packaging treatments maintained the quality and improved the shelf life of tomato fruit. IOP Conf. Ser. Earth Environ. Sci..

[15] Tzortzakis N., Chrysargyris A. (2017). Postharvest ozone application for the preservation of fruits and vegetables. Food Rev Int..

[16] Glowacz M., Rees D. (2016). The practicality of using ozone with fruit and vegetables. J. Sci. Food Agric..

[17] Pinto L., Caputo L., Quintieri L., de Candia S., Baruzzi F. (2017). Efficacy of gaseous ozone to counteract postharvest table grape sour rot. Food Microbiol..

[18] Ozkan R., Smilanick J.L., Karabulut O.A. (2011). Toxicity of ozone gas to conidia of *Penicillium digitatum*, *Penicillium italicum*, and *Botrytis cinerea* and control of gray mold on table grapes. Postharvest Biol. Technol..

[19] Lopes-Morais M., Alvinhäo J.E.O., Vilela-Franco D., Silva E.d.B., Dessimoni-Pinto N.A.V. (2015). Application of ozone aiming to keep the quality of strawberries using a low cost reactor. Rev Bras Frutic..

[20] Pinto L., Palma A., Cefola M., Pace B., D’Aquino S., Carboni C., Baruzzi F. (2020). Effect of modified atmosphere packaging (MAP) and gaseous ozone pre-packaging treatment on the physico-chemical, microbiological and sensory quality of small berry fruit. Food Packag. Shelf Life.

[21] Onopiuk A., Półtorak A., Moczkowska M., Szpicer A., Wierzbicka A. (2017). The impact of ozone on health-promoting, microbiological, and colour properties of *Rubus ideaus* raspberries. CyTA-J. Food.

[22] Piechowiak T., Antos P., Kosowski P., Skrobacz K., Józefczyk R., Balawejder M. (2019). Impact of ozonation process on the microbiological and antioxidant status of raspberries (*Rubus ideaeus* L.) during storage at room temperature. Agric Food Sci..

[23] Han Q., Gao H., Chen H., Fang X., Wu W. (2017). Precooling and ozone treatments affects postharvest quality of black mulberry (*Morus nigra*) fruits. Food Chem..

[24] Piechowiak T., Antos P., Józefczyk R., Kosowski P., Skrobacz K., Balawejder M. (2019). Impact of Ozonation Process on the Microbiological Contamination and Antioxidant Capacity of Highbush Blueberry (*Vaccinum corymbosum* L.) Fruit during Cold Storage. Ozone Sci. Eng..

[25] Barth M.M., Zhou C., Mercier J., Payne F.A. (1995). Ozone storage effects on anthocyanin content and fungal growth in blackberries. J. Food Sci..

[26] Pérez A.G., Sanz C., Ríos J.J., Olías R., Olías J.M. (1999). Effects of ozone treatment on postharvest strawberry quality. J. Agric. Food Chem..

[27] Tabakoglu N., Karaca H. (2018). Effects of ozone-enriched storage atmosphere on postharvest quality of black mulberry fruits (*Morus nigra* L.). LWT.

[28] Concha-Meyer A., Eifert J.D., Williams R.C., Marcy J.E., Welbaum G.E. (2015). Shelf Life Determination of Fresh Blueberries (*Vaccinium corymbosum*) Stored under Controlled Atmosphere and Ozone. Int. J. Food Sci..

[29] Sharpe D., Fan L., McRae K., Walker B., MaCKay R., Doucette C. (2009). Effects of ozone treatment on *Botrytis cinerea* and *Sclerotinia sclerotiorum* in relation to horticultural product quality. J. Food Sci..

[30] Chilosi G., Tagliavento V., Simonelli R. (2015). Application of ozone gas at low doses in the cold storage of fruit and vegetables. Acta Hortic..

[31] Instituto Ecuatoriano de Normalización (2010). INEN 2427:2010: Frutas Frescas. Mora. Requisitos.

[32] Brodowska A.J., Nowak A., Kondratiuk-Janyska A., Piątkowski M., Śmigielski K. (2017). Modelling the Ozone-Based Treatments for Inactivation of Microorganisms. Int. J. Environ. Res. Public Health.

[33] Pérez-Gallardo A., García-Almendárez B., Barbosa-Cánovas G., Pimentel-González D., Reyes-González L.R., Regalado C. (2015). Effect of starch-beeswax coatings on quality parameters of blackberries (*Rubus* spp.). J. Food Sci. Technol..

[34] Chen C., Zhang H., Zhang X., Dong C., Xue W., Xu W. (2020). The effect of different doses of ozone treatments on the postharvest quality and biodiversity of cantaloupes. Postharvest Biol. Technol..

[35] Allende A., Marín A., Buendía B., Tomás-Barberán F., Gil M.I. (2007). Impact of combined postharvest treatments (UV-C light, gaseous O_3_, superatmospheric O_2_ and high CO_2_) on health promoting compounds and shelf-life of strawberries. Postharvest Biol. Technol..

[36] Forney C.F., Song J., Fan L., Hildebrand P.D., Jordan M.A. (2003). Ozone and 1-MCP alter the postharvest quality of broccoli. J. Amer. Soc. Hort. Sci..

[37] Kumar P., Sethi S., Sharma R.R., Srivastav M., Varghese E. (2017). Effect of chitosan coating on postharvest life and quality of plum during storage at low temperature. Sci. Hortic..

[38] Joo M., Lewandowski N., Auras R., Harte J., Almenar E. (2011). Comparative shelf life study of blackberry fruit in bio-based and petroleum-based containers under retail storage conditions. Food Chem..

[39] Nayak S.L., Sethi S., Sharma R.R., Sharma R.M., Singh S., Singh D. (2020). Aqueous ozone controls decay and maintains quality attributes of strawberry (*Fragaria × ananassa* Duch.). J. Food Sci. Technol..

[40] Li H., Xiong Z., Gui D., Li X. (2019). Effect of aqueous ozone on quality and shelf life of Chinese winter jujube. J. Food Process. Preserv..

[41] Wieczynska J., Lovino R., Lamaj F., De Cillis M.F., Baser N., Ismaili K., Verrastro V., Tarricone L., Simeone V. (2015). Effect of O_3_ and high CO_2_ application during cold storage on quality of organic table grape (*Vitis vinifera* L. ‘Italia’). Acta Hortic..

[42] Horvitz S., Cantalejo M.J. (2010). The Effects of Gaseous Ozone and Chlorine on Quality and Shelf-life of Minimally Processed Red Pepper. Acta Hortic..

[43] Niveditha A., Pandiselvam R., Prasath V.A., Singh S.K., Gul K., Kothakota A. (2021). Application of cold plasma and ozone technology for decontamination of *Escherichia coli* in foods—A review. Food Control.

[44] Ali A., Yeoh W.K., Forney C., Siddiqui M.W. (2018). Advances in postharvest technologies to extend the storage life of minimally processed fruits and vegetables. Crit. Rev. Food Sci. Nutr..

[45] Emer Z., Akbas M.Y., Ozdemir M. (2008). Bactericidal activity of ozone against *E. coli* in whole and ground black peppers. J. Food Prot..

[46] Bialka K.L., Demirci A. (2007). Utilization of gaseous ozone for the decontamination of *Escherichia coli* O157:H7 and *Salmonella* on raspberries and strawberries. J. Food Prot..

[47] Akata I., Torlak E., Erci F. (2015). Efficacy of gaseous ozone for reducing microflora and foodborne pathogens on button mushroom. Postharvest Biol. Technol..

[48] Daş E., Candan-Gürakan G., Bayındırlı A. (2006). Effect of controlled atmosphere storage, modified atmosphere packaging and gaseous ozone treatment on the survival of *Salmonella enteritidis* on cherry tomatoes. Food Microbiol..

[49] Gibson K.E., Almeida G., Jones S.L., Wright K., Lee J.A. (2019). Inactivation of bacteria on fresh produce by batch wash ozone sanitation. Food Control.

[50] Jacques A.C., Zambiazi R.C., Ávila-Gandra E., Krumreich F., Rickies da Luz S., Ribeiro Galvão Machado M. (2015). Sanitization by Chlorine compounds and Ozone: Effect on Bioactive Compounds in Blackberry (*Rubus fruticosus*) cv. Tupy. Rev. Ceres.

[51] Vlassi E., Vlachos P., Kornaros M. (2018). Effect of ozonation on table grapes preservation in cold storage. J. Food Sci. Technol..

[52] Smilanick J.L., Margosan D.M., Mlikota F. (2002). Impact of ozonated water on the quality and shelf-life of fresh citrus fruit, stone fruit and table grapes. Ozone Sci. Eng..

[53] Brodowska A.J., Śmigielski K., Nowak A., Czyżowska A., Otlewska A. (2015). The Impact of Ozone Treatment in Dynamic Bed Parameters on Changes in Biologically Active Substances of Juniper Berries. PLoS ONE.

[54] Karakosta E.S., Karabagias I.K., Riganakos K.A. (2019). Shelf Life Extension of Greenhouse Tomatoes Using Ozonation in Combination with Packaging under Refrigeration. Ozone Sci. Eng..

[55] Alves H., Alencar E.R., Ferreira W.F.S., Silva C.R., Ribeiro J.L. (2019). Microbiological and physical-chemical aspects of strawberry exposed to ozone gas at different concentrations during storage. Braz. J. Food Technol..

[56] Liu Z., Li W., Zhai X., Li X. (2021). Combination of precooling with ozone fumigation or low fluctuation of temperature for the quality modifications of postharvest sweet cherries. J. Food Process. Preserv..

[57] Artés-Hernández F., Aguayo E., Artés F. (2004). Alternative atmosphere treatments for keeping quality of ‘Autumn seedless’ table grapes during long-term cold storage. Postharvest Biol. Technol..

[58] Horvitz S., Cantalejo M.J. (2014). Application of ozone for the postharvest treatment of fruits and vegetables. Crit. Rev. Food Sci. Nutr..

